# Impact of virtual consultations on quality of care in type 2 diabetes: a systematic review and narrative synthesis protocol

**DOI:** 10.1136/bmjopen-2023-082452

**Published:** 2024-11-02

**Authors:** Reham Aldakhil, Elena Lammila-Escalera, Benedict Hayhoe, Azeem Majeed, Geva Greenfield, Ana Luisa Neves

**Affiliations:** 1 Department of Primary Care and Public Health, Imperial College London, London, UK

**Keywords:** Telemedicine, Systematic Review, Diabetes and endocrinology, Health informatics, Quality in health care

## Abstract

**ABSTRACT:**

**Background:**

Around 463 million people globally have diabetes, of which over 90% have type 2 diabetes (T2D). Projections indicate an expected increase to 700 million by 2045. The COVID-19 pandemic accelerated digital health uptake, establishing virtual consultations as a feasible alternative to traditional in-person care. Despite promising preliminary evidence, a comprehensive review is needed to fully assess the impact of virtual consultations on diabetes care. This review aims to systematically evaluate the impact of remote consultations on the quality of care provided to persons with T2D, by mapping impacts against the six quality domains outlined by the National Academy of Medicine (NAM) (ie, patient-centeredness, effectiveness, efficiency, timeliness, equity and safety).

**Methods and analysis:**

PubMed/MEDLINE (Medical Literature Analysis and Retrieval System Online), COCHRANE Library, EMBASE (Excerpta Medica Database), CINAHL (Cumulative Index to Nursing and Allied Health Literature) and Web of Science will be searched for studies published between 2010 and 2024. Primary outcomes will include any quality measures pertaining to the NAM domains for adult patients accessing virtual consultations. The Cochrane Collaboration’s tool will be used to assess the quality of the randomised studies, and the Risk of Bias in Non-Randomised Studies of Interventions will be used for non-randomised studies. The findings will be summarised as a narrative synthesis. This systematic review protocol was registered with the International Prospective Register of Systematic Reviews on 15 November 2023 (registration number: CRD42023474219).

**Ethics and dissemination:**

This review will not include primary data and therefore does not require ethical approval. This protocol complies with the Preferred Reporting Items for Systematic Review and Meta-Analyses Protocols guidelines. Findings will be disseminated as academic publications and conference presentations and summarised into patient-led lay summaries.

**STRENGTHS AND LIMITATIONS OF THIS STUDY**

- Comprehensive inclusion criteria will encompass a variety of study designs, including randomised controlled trials, cluster randomised trials, quasi-experimental studies, case-control and cohort studies, cross-sectional analyses and cost-effectiveness studies, covering both primary and secondary care settings.
- The use of the National Academy of Medicine’s six domains of quality care offers a robust rationale for a comprehensive assessment of virtual care. An in-depth examination of the quality dimensions of virtual consultations (ie, telephone and video), ensures a holistic analysis of their implications on the quality of care delivered.
- The inclusion of diverse study designs may lead to heterogeneity in results, thus posing challenges to their synthesis.

## Introduction

 Around 463 million individuals worldwide currently have diabetes, representing 9.3% of people aged between 20 years and 79 years. Projections suggest an increase to 578 million (10.2%) by 2030, and further to 700 million (10.9%) by 2045.^1 2^ Type 2 diabetes (T2D) constitutes over 90% of those diagnosed with this long-term condition.^3^ T2D diagnosis is linked to the development of both microvascular and macrovascular complications, leading to significant psychological and physical distress for both patients and their caregivers.^4^ T2D also imposes a substantial burden on healthcare systems, as patients affected by this condition require regular monitoring and healthcare support.^5^

Traditionally, healthcare providers in primary and secondary care settings have monitored their patients with T2D primarily through in-person consultations. The COVID-19 pandemic has hastened the digital transformation in healthcare,^6 7^ with virtual consultations gaining significant attention as an alternative service delivery model.^8 9^ Postpandemic, this model of care persists, especially in the management of chronic conditions such as T2D. These consultations can be facilitated synchronously (in real-time, through videoconferencing or phone calls) or asynchronously, where patients upload their health information to a digital platform, with practitioners subsequently providing feedback or therapeutic recommendations at their convenience.^10^

Virtual consultations can enhance appointment attendance and decrease healthcare costs for T2D patients.^11 12^ Virtual consultations, particularly when synchronous, have also been effective in reducing HbA1c levels.^13 14^ While the empirical evidence supports the favourable outcomes and benefits of virtual consultations, they also have limitations.^15 16^ Concerns about virtual care include low technology literacy among older patients^17^,^19^ and potential reductions in personalised care.^6 20^ Additionally, while the adoption of virtual consultations has generally increased, uptake remains lower among older people, ethnic minorities and individuals from lower socioeconomic backgrounds,^11^ indicating inequities between patient groups.^21^ Additionally, virtual consultations may hinder the establishment of effective patient–provider relationships.^6 22 23^

These findings underscore the necessity of conducting a systematic review of the most recent evidence, particularly from the last 4 years, to grasp the true impact of virtual consultations.^24 25^ While virtual consultations were used before the pandemic, they were often limited in scope for specific conditions or in rural areas, and not extensively studied or regulated.^26 27^ The pandemic has significantly accelerated their use, which emphasises the need for more robust regulatory frameworks and policies to accommodate new digital practices.^28^ A comprehensive analysis is essential to guide healthcare policies and regulatory frameworks related to virtual consultations and shape licensure requirements, and privacy regulations,^7^ and further understand the implications on healthcare delivery, patient engagement and cost-efficiency.^29^

The aim of this review is to systematically evaluate the evidence regarding the impact of virtual consultations on the quality of care for T2D patients by mapping the impact against the six domains of the National Academy of Medicine (NAM), which was founded as the Institute of Medicine.^30^ NAM’s framework is often used to guide quality improvement initiatives and research, ensuring a structured, multifaceted assessment of the impact of digital interventions on care quality.^31^ The primary objective of this systematic review is to evaluate the impact of virtual consultations on the quality of care provided to adults with T2D and compare the outcomes with those of traditional in-person care to identify gaps in the current literature regarding the quality of care delivered through remote consultations.

## Methods

This review will adopt a comprehensive approach, exploring the existing literature to identify the impact of virtual consultations on the health outcomes of individuals with T2D, as compared with the traditional face-to-face model. It will follow the Preferred Reporting Items for Systematic Reviews and Meta-Analyses Protocols (PRISMA-P) checklist^32^ and will map the impacts against NAM’s six care quality domains: efficiency, effectiveness, safety, patient-centeredness, timeliness and equity (table 1). This systematic review protocol has been registered with the International Prospective Register of Systematic Reviews on 15 November 2023, (registration number: CRD42023474219), and was updated on 4 October 2024. The review is planned to start on 31 January 2024 and is expected to be completed by 31 March 2025.

**Table 1:** Mapping the impact of remote consultations for T2D patients based on the NAM* six domains analytical framework of quality of care

NAM domain	Description
Patient-centredness	Assess patient satisfaction with remote consultations, including both quantitative (eg, satisfaction scores) and qualitative data (eg, patient interviews and feedback).
Efficient	Analyse the impact of remote T2D care, for example, assessing cost-effectiveness, cost savings, resources utilisation and healthcare spending associated with remote care.
Safe	Assess the impact of remote consultations on medication errors and safety using quantitative measures such as error rates, safety incidents severity and mortality rates.
Effective	Evaluate the effectiveness of remote consultations on improving T2D patients’ health outcomes, specifically focusing on the treatment success rate, using quantitative measures (eg, HbA1c levels, blood pressure).
Timely	Timeliness of remote appointment availability, for example, quantified by the average waiting times for patients to access remote appointments, speed of follow-up after consultation.
Equitable	Access disparities across different sociodemographic statuses, ethnicity and geographical patient groups, for example, quantitative measures to identify disparities and assess progress towards equitable access.

NAMNational Academy of MedicineT2Dtype 2 diabetes

### Study selection criteria

The search strategy will be based on predefined eligibility criteria as highlighted in table 2.

**Table 2:** Predefined inclusion and exclusion criteria

	Inclusion	Exclusion
Population	Adult diagnosed with T2D, any gender.Any geographical location. Primary and secondary care.	Paediatric, Non-T2D (type 1, gestational diabetes).Tertiary or quaternary care settings. Social care centres or retail clinics.
Interventions	Real-time (synchronous) provider–patient remote consultation via videoconference or telephone	Only traditional in-person (face-to-face) consultations.Remote management of T2D by patients (eg, mHealth apps, remote or online log or tracking of glucose levels, diet or physical activities reported by patients not by primary healthcare providers, wearable devices).Remote solutions involve texting or emailing.
Comparisons	Face-to-face consultations	No face-to-face consultations comparison
Outcomes of interest	Studies reporting any quantitative measures related to NAM quality of care framework components.Effectiveness (eg, health outcomes).Equity (eg, disparities across different sociodemographic statuses, ethnicity and geographical patients’ groups).Efficiency (eg, cost of T2D care).Timeliness (eg, waiting time, appointment availability).Patient-centred (eg, patient satisfaction with the virtual consultation).Patient safety (eg, impact on medication errors).	Qualitative outcomes.Outcomes not related to NAM domains.
Study design	Randomised controlled trials, cluster randomised trials, quasi-experimental, case-control, cohort studies, cross sectional and cost-effectiveness.	Commentaries, conference papers, case reports, reviews.

NAMNational Academy of MedicineT2Dtype 2 diabetes

#### Population

Studies focusing on adults diagnosed with T2D will be included in this review. Studies that included all types of diabetes, that is, type 1 or gestational, will be included if providing a subgroup analysis for T2D patients, and these specific findings will be extracted. Studies of type 1 or gestational diabetes only will be excluded. Studies will be included if focusing on adults (mean age ≥18 years) that access primary or secondary care, with no geographical location restriction. Inclusion criteria are not restricted by language. Studies published in languages other than English will be included in the review.

#### Intervention

Real-time (synchronous) provider-patient remote consultation via videoconference or telephone. In cases where the intervention is a multicomponent, incorporating both synchronous and asynchronous elements, we will include such studies but will only consider the findings related to the synchronous components. Consultations between patients accessing services in primary or secondary care settings and healthcare providers such as general practitioners, medical doctors or specialists will be included.

#### Comparisons

The included studies will be required to have the traditional care of the same consultation delivered in person. In-person consultations will be standardised by typically in-person appointments focusing on face-to-face patient–provider communication and direct feedback. These will be compared with virtual ones with adjustment made for the modality of care.

#### Outcome measure

Primary outcomes will include any measures related to the NAM dimensions, that is, effectiveness (eg, health outcomes), equity (eg, disparities across different sociodemographic statuses, ethnicity, and geographical patients’ groups), efficiency (eg, cost of T2D care), timeliness (eg, waiting time and appointment availability), patient-centredness (eg, patient satisfaction with the virtual consultation) and patient safety (eg, impact on medication errors).

#### Type of studies

Studies will be included if they assess the impact of virtual consultations on adults with T2D, delivered by healthcare providers in primary or secondary care settings, using synchronous video or telephone consultation and reported outcomes of any of the NAM’s quality domains. A summary of the PICO (Population, Intervention, Comparison, and Outcome) and the study type to be included is presented in table 2. Inclusion criteria were not restricted by language. Non-English studies will be translated by professional services if selected for inclusion. Both peer-reviewed and grey literature will be included. Grey literature will be identified through Google Scholar and the criteria for inclusion will be consistent with the overall study selection criteria.

### Search strategy

The systematic search will be conducted on PubMed/MEDLINE, COCHRANE, EMBASE, CINAHL and Web of Science for studies published between 2010 and 2024. The rationale for choosing the period from 2010 to 2023 is that the adoption of digital health technologies began to accelerate around 2010; this period marks the beginning of widespread integration of electronic health records, telemedicine and other digital innovations in healthcare systems.^33 34^ This timeframe will include both prepandemic and postpandemic studies, which will help to understand how virtual consultations have developed. The search strategy will focus on three main concepts: virtual care, T2D and the NAM’s six domains of care quality.

Additionally, a screening of the reference lists of the relevant systematic reviews and sources of grey literature will be conducted. A detailed breakdown of the search terms and strings used in each database can be found in online supplemental 1.

### Screening and data extraction

Screening of the studies will be conducted independently by two investigators. We will use the PRISMA-P flow diagram to identify articles that meet the predefined eligibility criteria. Title and abstract screening will be conducted using Covidence software. The screening will be conducted in duplicate, with two reviewers independently assessing each study. For data extraction, one reviewer will extract the data, and the second reviewer will independently review the extracted data for accuracy and completeness. Any discrepancy will be resolved through discussion or by involving a third reviewer if necessary.

A standard data extraction form will be used to extract data from the included studies. The standard data extraction form will include fields for author, year, country, study design, sample size, participants, intervention, comparison/control, outcome measure, type of consultation, consultation description, domain(s) of quality care and key findings. Each author’s contribution to the data extraction process will be documented. If any data are missing or unclear in the included studies, we will contact the authors directly. Authors will be contacted up to three times over a 4-week period. If no response is received, we will evaluate the potential impact of the missing data. Studies with critical missing data that could significantly affect the analysis may be excluded. If a study is included despite incomplete data, the missing data will be noted in the limitation, and the risk of bias will be assessed accordingly.

### Risk of bias assessment

Bias assessment will be conducted using The Cochrane Collaboration’s tool, which categorises the risk of bias into three levels: ‘low risk’, ‘high risk’ or ‘unclear risk’. This tool comprises six domains of bias: selection bias, performance bias, detection bias, attrition bias, reporting bias and other bias. The Risk of Bias in Non-Randomised Studies of Interventions will be used for non-randomised studies, which will assess bias of confounding, selection of participants, classification of intervention, deviation from the intended interventions, missing data, measurement of outcomes and selection of reported results.^35^ Additionally, we will use the Mixed Methods Appraisal Tool to assess bias across the anticipated diverse range of study designs included in the review. Two independent reviewers will evaluate the risk of bias for each trial. In cases of disagreement, consensus will be reached through discussion, or if necessary, a third reviewer will arbitrate. A risk of bias narrative summary table and statement will be provided.

### Synthesis strategy

Due to the heterogeneity of outcome measures, it is unlikely that a meta-analysis will be possible. Therefore, the findings will be summarised using a narrative synthesis. To address the variability in study populations, intervensions and consultation modalities, results will be mapped across each of the NAM domains, with the key findings presented in a table for each domain. Descriptive statistics (means, medians and percentages) will be used to summarise quantitative data where appropriate, while subgroup analysis will be conducted based on consultation modalities (eg, video vs telephone), country, setting and patient age, if the data allow. When applicable, inferential statistics will be used to compare outcomes between virtual and in-person consultations. The potential for long-term integration of virtual consultations will also be assessed if reported by the included studies.

### Ethics and dissemination

This review will not include primary data and does not require ethical approval. This protocol complies with the PRISMA-P guidelines.

Findings will be disseminated as academic publications and conference presentations and summarised into patient-led lay summaries.

### Patient and public involvement

Patients did not contribute to the design of this systematic review protocol since it does not involve recruiting participants. However, patient partners will play a role in interpreting the results and in creating lay summaries and reports of the review findings. We will collaborate with the patient and public involvement and engagement theme at the Northwest London Applied Research Collaboration to ensure diverse representation in the review process.

## Discussion

The emergence of virtual care, accelerated by the COVID-19 pandemic, coupled with the projected increase in T2D cases, makes the systematic evaluation of the impact of virtual consultations a topical research area. Face-to-face consultations have been the bedrock of healthcare, offering personalised care, building patient–doctor rapport and facilitating in-depth discussions. However, remote consultations promise accessibility, convenience and a modern approach to healthcare delivery. The debate surrounding continuing the use of virtual consultation post-COVID-19 alongside traditional in-person consultations reflects the need to recognise the actual impact of virtual consultations and to form an evidence base to support their implementation. The use of NAM domains, a well-defined framework, provides a structured approach to evaluation. The application of this framework in other systematic reviews,^32^,^34^ where efficiency was measured by reduced appointment durations, effectiveness by improvement in HbA1c levels and patient centres through satisfaction surveys, demonstrates its robust utility in assessing the impact of digital interventions on care quality such as virtual consultations.

### Strengths and limitations

A robust framework is employed, using the NAM conceptual model, which is known for its structured approach in evaluating healthcare quality across six domains, thus allowing a comprehensive assessment of the intervention. The inclusive criteria will accommodate a range of study designs, participants and interventions, potentially increasing the generalisability of the findings. The use of automation tools for systematic reviews enhances the precision and comprehensiveness of literature search, leading to more robust and inclusive review. A potential limitation is the breadth of the NAM domains, the studies included may lack consistent definitions and measurements, leading to potential challenges in comparing outcomes.

### Potential implication for research, practice and policy

This review aims to identify the research gap within the virtual care, thereby directing future inquiries and contributing to the evidence base of virtual care models, particularly in the influence of remote technological advances. The findings will contribute to a better understanding of the role that virtual consultations play in managing long-term and prevalent conditions like T2D.

With T2D being a major contributor to the global healthcare burden, this review is expected to inform the optimisation of healthcare delivery by highlighting effective practice in virtual consultations for T2D management, and therefore, improve patient care by identifying effective care strategies. While not the focus of our current protocol, virtual consultations could also serve as a platform for AI-driven decision support systems, automating routine tasks and providing real-time analytics, ultimately contributing to more efficient and effective patient care.

Furthermore, the review will address access and equity issues in virtual delivery of care, providing insights into overcoming barriers for marginalised groups. Therefore, the findings can guide policy decisions, influence implementation strategies and inform the development and refinement of regulatory frameworks.

## supplementary material

10.1136/bmjopen-2023-082452 online supplemental file 1

## Data Availability

All data relevant to the study are included in the article or uploaded as supplementary information.
