# Supplementary material for: Impact of virtual consultations on quality of care in type 2 diabetes: a systematic review and narrative synthesis protocol
Source: BMJ Open. 2024 Nov 2;14(11):e082452. doi: 10.1136/bmjopen-2023-082452 (PMC11535670; doi:10.1136/bmjopen-2023-082452)
Supplement: online supplemental file 1 [file bmjopen-14-11-s001.docx]

***Supplementary I: Search terms and concepts***

| Database | Concept | Search Terms |
| --- | --- | --- |
| PubMed | Virtual Consultation | 1. (telemedicine or tele medicine or telehealth or tele health or telecare or tele care or teleconsult* or ((virtual* or remote* or telephon* or phone* or video* or online) adj3 (consult* or appointment*))) 2. telemedicine/ or remote consultation/ 3. 1 or 2 |
|  | Type 2 Diabetes | 1. (Diabetes Mellitus OR (Type 2 Diabetes Mellitus/ OR diabetes type 2 OR T2DM OR T2D OR Type two diabetes OR diabetes Type II OR diabet* OR Type 2 Diabet* OR T2DM OR T2D)) 2. (Non-Insulin-Dependent Diabetes Mellitus OR NIDDM OR non insulin-dependent diabetes OR NIDD OR ketosis resistant diabetes mellitus) 3. (Adult-Onset Diabetes OR Mature-Onset Diabetes OR maturity onset diabetes) 4. 4 or 5 or 6 |
|  | **NAM domains:**  Patient-centred | 1. (((experience* or satisfaction) adj4 (patient* or consumer* or client* or survey* or questionnaire*)) or PREM* or patient-reported experience measure* or patient-centred* or person-centred*) 2. Patient Satisfaction/ 3. 8 or 9 |
|  | Effectiveness | 1. Treatment Outcome/ or ((health or clinical* or treatment*) adj3 (outcome* or effective* or efficacy)) |
|  | Safety | 1. ((patient adj3 (safety or harm)) or misdiagnos* or safety manag* or (accident* adj2 prevent*) or error* or medication reconcil* or near miss*) 2. patient harm/ or patient safety/ or Diagnostic Errors/ 3. 12 or 13 |
|  | Efficiency | 1. (efficiency or economic* or cost* or expenditure* or charge* or (number adj3 appointment*) or (number adj3 admission*) or (number adj3 consultation*)) |
|  | Timeliness | 1. (wait* list* or wait* time* or timeliness) 2. Time-to-Treatment/ or Waiting Lists/ 3. 16 or 17 |
|  | Equity | 1. ((health* or health care or access) adj3 (equity or disparit* or inequit* or inequalit* or equality or gap)) 2. Health Equity/ 3. 19 or 20 |
|  |  | 1. 10 or 11 or 14 or 15 or 18 or 21 2. 3 and 7 and 22 |
|  |  | 1. limit 23 to yr="2010 - Current" |

| Database | Concept | Search Terms |
| --- | --- | --- |
| Web of Science | Virtual Consultation | 1. (telemedicine OR "tele medicine" OR telehealth OR "tele health" OR telecare OR "tele care" OR teleconsult* OR (virtual* OR remote* OR telephon* OR phone* OR video* OR online )) 2. (consult* OR appointment*) 3. 1 or 2 |
|  | Type 2 Diabetes | 1. ("Diabetes Mellitus" OR "Type 2 Diabetes Mellitus" OR "diabetes type 2" OR T2DM OR T2D OR "Type two diabetes" OR "diabetes Type II" OR diabet* OR "Type 2 Diabet*" OR T2DM OR T2D) 2. ("Non-Insulin-Dependent Diabetes Mellitus" OR NIDDM OR "non insulin-dependent diabetes" OR NIDD OR ketosis resistant diabetes mellitus) 3. ("Adult-Onset Diabetes" OR "Mature-Onset Diabetes" OR “maturity onset diabetes”) 4. 4 or 5 or 6 |
|  | **MOI domains:**  Patient-centred | 1. ((experience* OR satisfaction ) AND (patient* OR consumer* OR client* OR survey* OR questionnaire* )) OR PREM* OR "patient-reported experience measure*" OR patient-centred* OR person-centred* 2. ("Patient Satisfaction" OR "Satisfaction of Patients" OR "Patient Contentment" OR "Patient Experience" OR "Patient Perception" OR "Quality of Care" OR "Healthcare Quality" OR "Healthcare Experience") 3. 8 OR 9 |
|  | Effectiveness | 1. (health OR clinical* OR treatment* ) AND (outcome* OR effective* OR efficacy) |
|  | Safety | 1. ((patient AND (safety OR harm )) OR misdiagnos* OR "safety manag*" OR (accident* AND prevent* ) OR error* OR "medication reconcil*" OR "near miss*" 2. ("patient harm" OR "patient safety" OR "Diagnostic Errors") 3. 12 OR 13 |
|  | Efficiency | 1. (efficiency OR economic* OR cost* OR expenditure* OR charge* OR (number N3 appointment*) OR (number N3 admission*) OR (number N3 consultation*)) |
|  | Timeliness | 1. ("wait* list*" OR "wait* time*" OR timeliness) 2. ("Time-to-Treatment" OR "Waiting Lists") 3. 16 or 17 |
|  | Equity | 1. ((health* OR "health care" OR access ) AND (equity OR disparit* OR inequit* OR inequalit* OR equality OR gap )) 2. "Health Equity" 3. 19 or 20 |
|  |  | 1. 10 or 11 or 14 or 15 or 18 or 21 2. 3 and 7 and 22 |
|  |  | 1. limit 23 to yr="2010 - Current" |

***Supplementary I (Continue):***

***Supplementary I (Continue):***

| Database | Concept | Search Terms |
| --- | --- | --- |
| Cochrane | Virtual Consultation | 1. (telemedicine OR "tele medicine" OR telehealth OR "tele health" OR telecare OR "tele care" OR teleconsult* OR ((virtual* OR remote* OR telephon* OR phone* OR video* OR online ) NEAR/3 (consult* OR appointment* ))) 2. [mh ^telemedicine] OR [mh ^"remote consultation"] 3. #1 OR #2 |
|  | Type 2 Diabetes | 1. ("Diabetes Mellitus" OR ([mh ^"Type 2 Diabetes Mellitus"] OR "diabetes type 2" OR T2DM OR T2D OR "Type two diabetes" OR "diabetes Type II" OR diabet* OR ("Type 2" NEXT Diabet*) OR T2DM OR T2D )) 2. ("Non-Insulin-Dependent Diabetes Mellitus" OR NIDDM OR "non insulin-dependent diabetes" OR NIDD OR ketosis resistant diabetes mellitus) 3. ("Adult-Onset Diabetes" OR "Mature-Onset Diabetes" OR “maturity onset diabetes”) 4. #4 OR #5 OR #6 |
|  | **NAM domains:**  Patient-centred | 1. (((experience* OR satisfaction ) NEAR/4 (patient* OR consumer* OR client* OR survey* OR questionnaire* )) OR PREM* OR ("patient-reported experience" NEXT measure*) OR patient-centred* OR person-centred* ) 2. [mh ^"Patient Satisfaction"] 3. #8 OR #9 |
|  | Effectiveness | 1. [mh ^"Treatment Outcome"] OR ((health OR clinical* OR treatment* ) NEAR/3 (outcome* OR effective* OR efficacy )) |
|  | Safety | 1. ((patient NEAR/3 (safety OR harm )) OR misdiagnos* OR ("safety" NEXT manag*) OR (accident* NEAR/2 prevent* ) OR error* OR ("medication" NEXT reconcil*) OR ("near" NEXT miss*) ) 2. [mh ^"patient harm"] OR [mh ^"patient safety"] OR [mh ^"Diagnostic Errors"] 3. #12 OR #13 |
|  | Efficiency | 1. (efficiency OR economic* OR cost* OR expenditure* OR charge* OR (number NEAR/3 appointment* ) OR (number NEAR/3 admission* ) OR (number NEAR/3 consultation* )) |
|  | Timeliness | 1. ((wait* NEXT list*) OR (wait* NEXT time*) OR timeliness ) 2. [mh ^Time-to-Treatment] OR [mh ^"Waiting Lists"] 3. #16 OR #17 |
|  | Equity | 1. ((health* OR "health care" OR access ) NEAR/3 (equity OR disparit* OR inequit* OR inequalit* OR equality OR gap )) 2. [mh ^"Health Equity"] 3. #19 OR #20 |
|  |  | 1. #10 OR #11 OR #14 OR #15 OR #18 OR #21 |
|  |  | 1. #3 AND #7 AND #22 limit: to "January 2010 -Current" |

***Supplementary I (Continue):***

| Database | Concept | Search Terms |
| --- | --- | --- |
| CINAHL via EBSCO | Virtual Consultation | 1. (telemedicine OR "tele medicine" OR telehealth OR "tele health" OR telecare OR "tele care" OR teleconsult* OR (virtual* AND (consult* OR appointment*))) 2. (TX telemedicine OR TX "remote consultation") 3. 1 or 2 |
|  | Type 2 Diabetes | 1. ("Diabetes Mellitus" or "Type 2 Diabetes Mellitus" or "diabetes type 2" or "T2DM" or "T2D" or "Type two diabetes" or "diabetes Type II" or "Type 2 Diabet*").mp. [mp=title, abstract, full text, caption text] 2. ("Non-Insulin-Dependent Diabetes Mellitus" OR NIDDM OR "non insulin-dependent diabetes" OR NIDD OR ketosis resistant diabetes mellitus) 3. ("Adult-Onset Diabetes" OR "Mature-Onset Diabetes" OR “ maturity onset diabetes”) 4. 4 or 5 or 6 |
|  | **MOI domains:**  Patient-centred | 1. (experience* OR satisfaction) AND (patient* OR consumer* OR client* OR survey* OR questionnaire*) OR "patient-reported experience measures" OR patient-centred OR person-centred 2. ("Patient Satisfaction" OR "Satisfaction of Patients" OR "Patient Contentment" OR "Patient Experience" OR "Patient Perception" OR "Quality of Care" OR "Healthcare Quality" OR "Healthcare Experience") AND (TI AB). 3. 8 or 9 |
|  | Effectiveness | 1. "Treatment Outcome" OR (health OR clinical* OR treatment*) AND (outcome* OR effective* OR efficacy) |
|  | Safety | 1. (patient AND (safety OR harm)) OR misdiagnos* OR (safety AND management) OR (accident* AND prevention) OR error* OR (medication AND reconciliation) OR "near miss" 2. (TI "patient harm" OR AB "patient harm" OR MH "Patient Harm") OR   (TI "patient safety" OR AB "patient safety" OR MH "Patient Safety") OR  (TI "Diagnostic Errors" OR AB "Diagnostic Errors" OR MH "Diagnostic Errors")   1. 12 or 13 |
|  | Efficiency | 1. (efficiency OR economic* OR cost* OR expenditure* OR charge* OR (number N3 appointment*) OR (number N3 admission*) OR (number N3 consultation*)) |
|  | Timeliness | 1. ("waiting list*" OR "waiting time*" OR timeliness) AND (TI AB) 2. ("Time-to-Treatment" OR "Waiting Lists") AND (TI AB TX) 3. 16 or 17 |
|  | Equity | 1. (health* OR "health care" OR access) AND (equity OR disparity* OR inequity* OR inequality* OR equality OR gap) 2. "Health Equity" AND (TI "Health Equity" OR AB "Health Equity") 3. 19 or 20 |
|  |  | 1. 10 or 11 or 14 or 15 or 18 or 21 2. 3 and 7 and 22 |
|  |  | 1. Limitation (2010_current) |

***Supplementary I (Continue):***

| Database | Concept | Search Terms |
| --- | --- | --- |
| EMBASE | Virtual Consultation | 1. (telemedicine OR "tele medicine" OR telehealth OR "tele health" OR telecare OR "tele care" OR teleconsult* OR ((virtual* OR remote* OR telephon* OR phone* OR video* OR online) NEAR/3 (consult* OR appointment*))) 2. 'telemedicine'/exp OR 'remote consultation'/exp 3. #1 OR #2 |
|  | Type 2 Diabetes | 1. ("diabetes mellitus" OR 'diabetes mellitus type 2'/exp OR "diabetes type 2" OR T2DM OR T2D OR "type two diabetes" OR "diabetes type II" OR diabet* OR ("type 2" NEXT diabet*)) 2. ("non-insulin-dependent diabetes mellitus" OR NIDDM OR "non insulin-dependent diabetes" OR NIDD OR "ketosis resistant diabetes mellitus") 3. ("adult-onset diabetes" OR "mature-onset diabetes" OR "maturity onset diabetes") 4. #4 OR #5 OR #6 |
|  | **MOI domains:**  Patient-centred | 1. (((experience* OR satisfaction) NEAR/4 (patient* OR consumer* OR client* OR survey* OR questionnaire*)) OR PREM* OR ("patient-reported experience" NEXT measure*) OR patient-centred* OR person-centred*) 2. 'patient satisfaction'/exp 3. #8 OR #9 |
|  | Effectiveness | 1. 'treatment outcome'/exp OR ((health OR clinical* OR treatment*) NEAR/3 (outcome* OR effective* OR efficacy)) |
|  | Safety | 1. ((patient NEAR/3 (safety OR harm)) OR misdiagnos* OR ("safety" NEXT manag*) OR (accident* NEAR/2 prevent*) OR error* OR ("medication" NEXT reconcil*) OR ("near" NEXT miss*)) 2. 'patient harm'/exp OR 'patient safety'/exp OR 'diagnostic error'/exp 3. #12 OR #13 |
|  | Efficiency | 1. (efficiency OR economic* OR cost* OR expenditure* OR charge* OR (number NEAR/3 appointment*) OR (number NEAR/3 admission*) OR (number NEAR/3 consultation*)) |
|  | Timeliness | 1. ((wait* NEXT list*) OR (wait* NEXT time*) OR timeliness) 2. 'time to treatment'/exp OR 'waiting list'/exp 3. #16 OR #17 |
|  | Equity | 1. ((health* OR "health care" OR access) NEAR/3 (equity OR disparit* OR inequit* OR inequalit* OR equality OR gap)) 2. 'health equity'/exp 3. #19 OR #20 |
|  |  | 1. #10 OR #11 OR #14 OR #15 OR #18 OR #21 2. #3 AND #7 AND #22 |
|  |  | Limit: 2010_current |
