# Supplementary material for: Impact of virtual consultations on quality of care in type 2 diabetes: a systematic review and narrative synthesis protocol
Source: BMJ Open. 2024 Nov 2;14(11):e082452. doi: 10.1136/bmjopen-2023-082452 (PMC11535670; doi:10.1136/bmjopen-2023-082452)
Supplement: Supplementary file 2 [file bmjopen-2023-082452.reviewer_comments.pdf]

PEER REVIEW HISTORY

BMJ Open publishes all reviews undertaken for accepted manuscripts. Reviewers are asked to complete a checklist review form and are provided with free text boxes to elaborate on their assessment. These free text comments are reproduced below.

ARTICLE DETAILS

Title (Provisional)

The Impact of Virtual Consultations on Quality of Care in Type 2 Diabetes: A Systematic Review and Narrative Synthesis Protocol

Authors

Aldakhil, Reham; Lammila-Escalera, Elena; Hayhoe, Benedict; Majeed, Azeem; Greenfield, Geva; Neves, Ana Luisa

---

VERSION 1 - REVIEW

**Reviewer**	**1**
**Name**	**Tsujii, Satoru**
**Affiliation**	**Tenri Hospital, Endocrinology**
**Date**	**31-Jan-2024**
**COI**	**None.**

The authors are searching the study reports published between 2010 and 2023 to evaluate the impact of synchronous remote consultations on the quality of care for persons with type 2 diabetes in the NAM's six quality domains. The rationale of this attempt is very well understandable. The authors could address the points written below.

The authors seemed not to specify report characteristics like language and publication status to be used as criteria for eligibility for the review.

Though the length of the intervention period might be varied in each study searched from the database, the durability or sustainability of virtual consultations should be challenging since persons need lifetime consultations in a chronic condition like type 2 diabetes. What do the authors state opinion and process in this protocol?

In the context of the progressively evolving artificial intelligence, how the real-time virtual consultations with healthcare providers would pave the way or contribute to the next stage?

**Reviewer**	**2**
**Name**	**Rohloff, Peter**

Affiliation Center for Research in Indigenous Health, Wuqu' Kawoq
Date 05-Apr-2024
COI none

This is a good protocol paper. I have only one comment. Since it seems to include all geographic locations and country income levels, it should also specify whether abstracts and articles not in English will be included (they should be, unless a very strong justification could be made).

Reviewer 3
Name Alvarez-Villalobos, Neri
Affiliation Universidad Autonoma de Nuevo Leon, Subdireccion de Investigación
Date 23-Apr-2024
COI none to declare

First of all, I would like to congratulate the research team. As mentioned, diabetes is a serious health problem known to affect people's health in various ways and even decrease life expectancy. Due to this, your work is extremely valuable and important. While many of us rely on telemedicine, we don't truly understand the real impact of virtual consultations.

Through this systematic review protocol, the authors aim to conduct a study to narratively synthetize the results of various articles of different designs about the impact of remote consultation on quality care in adults with type 2 diabetes. The domains of quality care are safety, effectiveness, patient-centeredness, efficiency, timeliness, and equity.

After a critical evaluation based on the Reporting checklist for protocol of a systematic review and meta analysis (PRISMA) and trying to prevent sources bias in the domains reported on the tool "A MeaSurement Tool to Assess systematic Reviews" (AMSTAR-2), several concerns arise. The authors may want to consider the following recommendations to improve the quality of their protocol:

Regarding the authors:

• Add the physical mailing address of the correspondence author.

• Add the email address of all authors.

Regarding the abstract:

• Although authors did provide the PROSPERO registration number, according to PRISMA-P guidelines, for improving transparency and avoiding possible replication of reviews, a more detailed registration should be provided, for example: "Our systematic review protocol was

registered with the International Prospective Register of Systematic Reviews (PROSPERO) on 11 July 2011 and was last updated on 19 January, 2012 (registration number: CRD42011001410)".

Regarding the introduction:

• The six domains of the National Academy of Medicine (NAM) offer a structured approach to evaluating the impact of interventions on care quality. However, it's important to consider whether these domains cover all relevant aspects for evaluating the quality of care for T2D patients through virtual consultations. Additional domains such as accessibility, adherence, quality of life, and cost-effectiveness could be beneficial to include for a more comprehensive evaluation. While these additional domains are implied, explicitly mentioning them would ensure a thorough assessment of the impact of virtual consultations on the quality of care for T2D patients.

• Include the data on how virtual consultations were conducted before the pandemic, as studies from 2010 are included in inclusion criteria, please highlight the main changes, if identified.

• Provide an explicit statement of objectives or questions addressed by the review.

Regarding the methods:

• Although authors did provide the PROSPERO registration number, according to PRISMA-P guidelines, for improving transparency and avoiding possible replication of reviews, a more detailed registration should be provided, for example: "Our systematic review protocol was registered with the International Prospective Register of Systematic Reviews (PROSPERO) on 11 July 2011 and was last updated on 19 January, 2012 (registration number: CRD42011001410)" .

• "Study selection criteria" goes before "search strategy."

• If possible include Scopus on the data bases you will use.

• Please describe where and how would you search for grey literature and what will be the criteria to include.

• Please describe how the in-person consultation will be standardized to be able to compare it with virtual consultations.

• Are the control groups allowed to have some kind of remote consultation? State in the criteria.

• Outcome measure: Specify the how positive health outcomes are going to be measured. HbA1c levels, weight, symptoms, complications?

• Please specify how patient satisfaction and medical error will be measured.

• In the search strategy, is there any reason to just use the last 10 years? Please justify it a little.

• We suggest using the time frame for the search change to 2013 up the date the search strategy is conducted.

• Expand the term diabetes on the search strategy using the following terms: "maturity onset diabetes mellitus" OR "diabetes mellitus, non insulin dependent" OR "diabetes mellitus, non−insulin−dependent" OR "diabetes mellitus, type 2" OR "diabetes mellitus, type II" OR "diabetes, adult onset” OR "dm 2" OR "insulin independent diabetes" OR "insulin independent diabetes mellitus" OR "ketosis resistant diabetes mellitus" OR "maturity onset diabetes" OR "maturity onset diabetes mellitus"

• Add more specific time limits in the search strategy in order to attain replicability. E.g. while using Cochrane there is the option to limit the search with months.

• Page 6 “Screening and data extraction”: Expand the explanation of how you will manage the reports. What software will you be using?

• Page 6 “Screening and data extraction”: Describe if the data extraction will be made in duplicate and how discrepancies will be solved. Will be the same methodology as with the screening?

• Add a subtitle describing how you will handle with “Missing data”. Are you going to contact the authors? How many times? How long will you wait for a response?

• On the screening and data extraction: As recommended in guidelines, for

enhancing reproducibility, authors should provide more detail on the "standard data extraction form" that investigators would use and specify author's contributions.

• Regarding the risk of bias assessment, as the study would include different types of studies, would the investigators only use the "Cochrane´s collaboration tool" and ROBINS? As authors would also include different types of non interventional studies (case-control, cohort studies, cross sectional and cost-effectiveness), the bias assessment tools according to the study designs should be cited.

• On the screening and data extraction: As recommended in guidelines, for

enhancing reproducibility, authors should provide more detail on the “standard data extraction form” that investigators would use and specify author’s contributions.

• Page 9: Expand lines 37 and 38: “The impact of each element within the domains will be statistically analyzed…” As a meta-analysis is not being planned, I got confused to what this analysis means and whether it is appropriate. Statistical analysis plan should be sufficiently described to enable reproduction and demonstrate the positive or negative impact of the variables.

• On page 4, it is stated that information from the last four years is particularly relevant to the study, and maybe due to the pandemic. However, I believe that data from previous years (early 2010 ÃÅs) could strengthen the argument that virtual consultations were not extensively studied or regulated. Incorporating this historical context could strengthen your

discussion and help justify the importance of regulating virtual consultations, especially since the pandemic has significantly increased their use.

• It is unclear whether the six domains of the National Academy of Medicine (NAM) will be evaluated and retrieved as quantitative or qualitative data. I couldn't find a specific method for this on the Agency for HealthCare Research and Quality page. This is particularly important because page nine mentions, 'The impact of each element within the domains will be statistically analyzed to demonstrate whether a negative, positive, or no impact is indicated.' Additionally, Table 1 indicates that domains like 'effective' and 'equitable' will be evaluated using quantitative measures. It is essential to more precisely define the domains, as Table 1 contains several instances of phrases like 'such as.' Remember, your analysis plan is crucial, so presenting your variables could also be helpful, although this will be discussed later on.

• It is mentioned that the heterogeneity of outcome measures makes a meta-analysis unlikely. It would be helpful to explain what will be done to address this heterogeneity in the narrative analysis.

• As mentioned before a potential limitation is the lack of definition of the NAM domains; the included studies may lack consistent definitions and measures, leading to possible challenges in comparing results so it would be helpful to explain what will be done to address this.

• Please provide a description of any subgroup analyses intended for the study. Subgroups may include analysis by year, age group of the patients, tyme from diagnosis, health status, pandemic time-lapse, use of artificial intelligence, new technologies, etc.

• Justify the absence of the GRADE framework.

• It would be beneficial to clarify how patient partners will be selected and involved in interpreting the results.

• There is contradiction in the concepts of synchronous and asynchronous times in Table 2 and in the intervention section, handle the explanation of this correctly.

Regarding the funding

• Page 21 support, sources: It was stated that no financial support was received. However, in page 11 lines 20-22 it is mentioned that the work is supported by the NIHR.

• Page 11 lines 28-32 should go to the “declaration of interests” subtitle.

Regarding the discussion:

• The discussion section helps us to contrasts the results of different studies with the results of the present one, although authors cite on the Introduction studies that helps us understand the magnitude of the issue, it would be helpful to also include similar studies that have assessed the quality of care of consultations using the NAM ́s quality domains to understand how are they measured.

• You mention the use of automatation tools as an strenght of the systematic review, but in the methods there is no description of which and how automatation tools will be utilized. Please provide a detailed description of this.

Extra

• Please check for grammatical and typographical errors.

VERSION 1 - AUTHOR RESPONSE

**Reviewer 1**	
The authors seemed not to specify report characteristics like language and publication status to be used as criteria for eligibility for the review.	We have now explicitly stated the language and publication status criteria in the manuscript to clarify the review process, The edited version of the manuscript now reads as follows: "Inclusion criteria were not restricted by language. Non-English studies will be translated by professional services if selected for inclusion. Both peer-reviewed and grey literature will be included. Grey literature will be identified through Google Scholar and the criteria for inclusion will be consistent with the overall study selection criteria."
Though the length of the intervention period might be varied in each study searched from the database, the durability or sustainability of virtual consultations should be challenging since persons need lifetime consultations in a chronic condition like type 2 diabetes. What do the authors state opinion and process in this protocol?	Thank you for highlighting the important of the durability or sustainability of virtual consultations for chronic conditions like Type 2 Diabetes and we agree that long-term sustainability is crucial.<br>We updated the methods section to highlight that, in our narrative synthesis, we will evaluate the effectiveness of virtual consultations and discuss their potential for long term integration into type 2 diabetes care management. Results will include long-term patient outcomes and healthcare system integration. The edited version of the manuscript now reads as follows:<br>"The impact of each element within the domains will be statistically analysed to demonstrate whether a negative, positive, or no impact is indicated. The potential for long-term integration and sustainability of virtual consultations will also be assessed, if reported by the included studies."
In the context of the progressively evolving artificial intelligence, how the real-time virtual consultations with healthcare providers would pave the way or contribute to the next stage?	Thank you for the insightful comment. Virtual consultations could serve as a platform for deploying AI-driven decision support systems, automating routine tasks, and providing real-time analytics, ultimately contributing to more efficient and effective patient care. While this is not within the focus of our current protocol, we introduce this relationship between AI and virtual consultations in our potential implications section.
**Reviewer 2**	
Since it seems to include all geographic locations and country income levels, it should also specify whether abstracts and articles not in English will be included (they should be, unless a very strong justification could be made).	Thank you for your comment. We confirm that our review will include abstracts and articles from all geographic locations and country income levels, regardless of the language of publication. A clarification of this was added in the selection criteria as following: "Inclusion criteria are not restricted by

	language. Studies published in languages other than English will be included in the review”
**Reviewer 3**	
**Regarding the authors:**<br>• Add the physical mailing address of the correspondence author.<br>• Add the email address of all authors.	We have added the physical mailing address of the corresponding author and included the email addresses for all authors in the manuscript as requested.
**Regarding the abstract:**<br>• Although authors did provide the PROSPERO registration number, according to PRISMA-P guidelines, for improving transparency and avoiding possible replication of reviews, a more detailed registration should be provided, for example: “Our systematic review protocol was registered with the International Prospective Register of Systematic Reviews (PROSPERO) on 11 July 2011 and was last updated on 19 January, 2012 (registration number: CRD42011001410)”.	Thank you for your suggestion. We agree that providing a more detailed registration statement improves transparency. We have updated the abstract to include additional details about the PROSPERO registration, including the registration date and any updates, as following:<br>“This systematic review protocol was registered with the International Prospective Register of Systematic Reviews (PROSPERO) on 15 November 2023, registration number: (CRD42023474219).”
**Regarding the introduction:**<br>**1.** The six domains of the National Academy of Medicine (NAM) offer a structured approach to evaluating the impact of interventions on care quality. However, it's important to consider whether these domains cover all relevant aspects for evaluating the quality of care for T2D patients through virtual consultations. Additional domains such as accessibility, adherence, quality of life, and cost-effectiveness could be beneficial to include for a more comprehensive evaluation. While these additional domains are implied, explicitly mentioning them would ensure a thorough assessment of the impact of virtual consultations on the quality of care for T2D patients.	**1.** Thank you for your thoughtful comments.t The six domains of NAM encompass the relevant aspects for evaluating the quality of care for T2D patients through virtual consultations, as explained below in further detail. Specifically, equity covers accessibility, as supported by literature addressing that equitable healthcare includes overcoming barriers to access. We will capture adherence as part of both effectiveness (achieving effective healthcare outcomes), and patient-centeredness (as tailored, patient-centred care might impact adherence as an outcome). Outcomes pertaining to quality of life will be included in as clinical effectiveness outcomes as well. Efficiency addresses cost-effectiveness outcomes, as demonstrated in studies evaluating the economic impact of healthcare delivery modality.
**2.** Include the data on how virtual consultations were conducted before the pandemic, as studies from 2010 are included in inclusion criteria, please highlight the main changes, if identified.	**2.** Thank you for the suggestion. A detailed description of the virtual consultation intervention for each study, both before and after the pandemic, is provided in data extraction form table. The table includes a breakdown of how virtual consultations were conducted across different time periods.
**3.** Provide an explicit statement of objectives or questions addressed by the review.	**3.** We have included an explicit statement of the objectives and research questions addressed by the review.
**Regarding the methods:**	Thank you for your thorough and insightful comments. We have revised our manuscript to address the points you raised.
**1.** Although authors did provide the PROSPERO registration number, according	**1.** Thank you for your suggestion. We have added more detailed information regarding the PROSPERO registration, as

to PRISMA-P guidelines, for improving transparency and avoiding possible replication of reviews, a more detailed registration should be provided, for example: “Our systematic review protocol was registered with the International Prospective Register of Systematic Reviews (PROSPERO) on 11 July 2011 and was last updated on 19 January, 2012 (registration number: CRD42011001410)” .	following: “This systematic review protocol has been registered with the International Prospective Register of Systematic Reviews (PROSPERO), on 15 November 2023, (registration number: CRD42023474219.”
**2.** “Study selection criteria” goes before “search strategy.”	**2.** We have now reordered the "Study Selection Criteria" and "Search Strategy" sections to align with your recommendation.
**3.** If possible include Scopus on the data bases you will use.	**3.** After careful consideration, we decided not to include Scopus as it may introduce significant overlap with MEDLINE/Embase, which are already covered in our search. Our current selection of databases PubMed/MEDLINE, Cochrane, EMBASE, CINAHL, and Web of Science provide comprehensive coverage to capture the necessary literature for this review.
**4.** Please describe where and how would you search for grey literature and what will be the criteria to include.	**4.** We have expanded our description of how and where grey literature will be searched, and criteria for inclusion.
**5.** Please describe how the in-person consultation will be standardized to be able to compare it with virtual consultations.	**5.** We have described how in-person consultations will be standardised to ensure compatibility, as following: “In-person consultations will be standardised by typically in-person appointment focusing on: face-to-face patient-provider communication and direct feedback. These will be compared with virtual ones with adjustment made for the modality of care”.
**6.** Are the control groups allowed to have some kind of remote consultation? State in the criteria.	**6.** We have clarified this in the manuscript: “Included studies will required to have received traditional in person consultations”.
**7.** Outcome measure: Specify the how positive health outcomes are going to be measured. HbA1c levels, weight, symptoms, complications?	**7.** Thank you for raising this point. We have intentionally kept the outcome measures broad to ensure we capture as much data as possible from the existing literature, as far as it includes the broad quality domains. This allows the inclusion of diverse measures.
**8.** Please specify how patient satisfaction and medical error will be measured.	**8.** As mentioned in our previous response, at this stage, we have chosen not to pre-define the specific metrics for patient-satisfaction and medical error to ensure that we capture the full range of data available in the included studies. Anticipated metrics may include, but are not limited to, standardised patient satisfaction surveys, patient reported experience measures, and recorded errors or adverse events.
**9.** In the search strategy, is there any reason to just use the last 10 years? Please justify it a little.<br>• We suggest using the time frame for the search change to 2013 up the date the search strategy is conducted.	**9.** Thank you for the suggestion. We decided to retain the search period from January 2010 and December 2023. The rational for this period is that the adoption of digital health technologies begun to accelerate around 2010, and the timeframe will include both pre- and post-pandemic studies, which will help to understand how virtual consultations have develop, we have provided this justification in the search strategy section of the protocol.

**10.** Expand the term diabetes on the search strategy using the following terms: "maturity onset diabetes mellitus" OR "diabetes mellitus, non insulin dependent" OR "diabetes mellitus, non−insulin−dependent" OR "diabetes mellitus, type 2" OR "diabetes mellitus, type II" OR "diabetes, adult onset" OR "dm 2" OR "insulin independent diabetes" OR "insulin independent diabetes mellitus" OR "ketosis resistant diabetes mellitus" OR "maturity onset diabetes" OR "maturity onset diabetes mellitus"	**10.** We have expanded the diabetes search terms to include the additional ones as suggested.
**11.** Add more specific time limits in the search strategy in order to attain replicability. E.g. while using Cochrane there is the option to limit the search with months.	**11.** Our search period is set from January 2010 to December 2023, we added a clarification on this time frame in the search strategy.
**12.** Page 6 “Screening and data extraction”: Expand the explanation of how you will manage the reports. What software will you be using?<br>• Page 6 “Screening and data extraction”: Describe if the data extraction will be made in duplicate and how discrepancies will be solved. Will be the same methodology as with the screening?	**12.** Screening and data extraction will be conducted using Covidence software. We have expanded this explanation in the manuscript as following: **“**Screening will be conducted using Covidence software The screening will be conducted in duplicate, with two reviewers independently assessing each study. For data extraction, one reviewer will extract the data, and the second reviewer will independently review the extracted data for accuracy and completeness. Any discrepancy will be resolved through discussion or by involving a third reviewer if necessary”.
**13.** Add a subtitle describing how you will handle with “Missing data”. Are you going to contact the authors? How many times? How long will you wait for a response?	**13.** We have expanded on handling the missing data in the manuscript as following: “If any data are missing or unclear in the included studies, we will contact the authors directly. Authors will be contacted up to three times over a four-week period. If no response is received, the study will be included, but the missing data will be noted in the discussion and limitation.**”**
**14.** On the screening and data extraction: As recommended in guidelines, for enhancing reproducibility, authors should provide more detail on the "standard data extraction form" that investigators would use and specify author's contributions.	**14.** We have now provided more details on the standard data extraction form and specified author contributions, as following: “The standard data extraction form will include fields for Author, Year, Country, Study Design, Sample Size, Participants, Intervention, Comparison/Control, Outcomes Measure, Type of Consultation, Consultation Description, Domain(s) of Quality Care, Key Findings. Each author’s contribution to the data extraction process will be documented.”.
**15.** Regarding the risk of bias assessment, as the study would include different types of studies, would the investigators only use the "Cochrane´s collaboration tool" and ROBINS? As authors would also include different types of non interventional studies (case-control, cohort studies, cross sectional and cost-effectiveness), the bias assessment tools according to the study designs should be cited.	**15.** Thank you for highlighting this. In addition to Cochrane Collaboration’s tool for randomised studies, we will utilise the Mixed Methods Appraisal Tool (MMAT) to assess bias across diverse range of study designs included in the review. We have clarified that we will be also using MMAT tool in the updated manuscript. The updated version is now reads as: “Additionally, we will utilise the Mixed Methods Appraisal Tool (MMAT) to assess bias across the anticipated diverse range of study designs included in the review.”

**16.** Page 9: Expand lines 37 and 38: "The impact of each element within the domains will be statistically analyzed…" As a meta-analysis is not being planned, I got confused to what this analysis means and whether it is appropriate. Statistical analysis plan should be sufficiently described to enable reproduction and demonstrate the positive or negative impact of the variables.	**16.** We have expanded and clarified this in the statistical analysis plan as following: "The impact of each element within the NAM domains will be statistically analysed using descriptive statistics and when applicable, inferential statistics to compare outcomes between virtual and in-person consultations. This analysis will allow us to determine whether a positive, negative, or neutral impact is indicated across the domains. However, given the anticipated heterogeneity of outcomes, the analysis will be narrative with quantitative data presented where appropriate using descriptive statistics such as, means, medians, and percentages.".
**17.** On page 4, it is stated that information from the last four years is particularly relevant to the study, and maybe due to the pandemic. However, I believe that data from previous years (early 2010 ÃÅs) could strengthen the argument that virtual consultations were not extensively studied or regulated. Incorporating this historical context could strengthen your discussion and help justify the importance of regulating virtual consultations, especially since the pandemic has significantly increased their use.	**17.** Thank you for highlighting this argument. We have expanded to address this discussion on virtual consultations before the pandemic and their development and added the supporting references, as following:<br>"While virtual consultations were utilised before the pandemic, they were often limited in scope for specific conditions or in rural areas, and not extensively studied or regulated. The pandemic has significantly accelerated their use, which emphasising the need for more robust regulatory frameworks and policies to accommodate new digital practices."
**18.** It is unclear whether the six domains of the National Academy of Medicine (NAM) will be evaluated and retrieved as quantitative or qualitative data. I couldn't find a specific method for this on the Agency for HealthCare Research and Quality page. This is particularly important because page nine mentions, 'The impact of each element within the domains will be statistically analyzed to demonstrate whether a negative, positive, or no impact is indicated.' Additionally, Table 1 indicates that domains like 'effective' and 'equitable' will be evaluated using quantitative measures. It is essential to more precisely define the domains, as Table 1 contains several instances of phrases like 'such as.' Remember, your analysis plan is crucial, so presenting your variables could also be helpful, although this will be discussed later on.	**18.** Thanks for bringing up this matter. The evaluation of the NAM domains will involve both quantitative and qualitative data. Domains such as effectiveness will be assessed using quantitative measures, including clinical outcomes (e.g., HbA1c levels, blood pressure, complications rate). Efficiency will also be assessed through quantitative metrics, such as cost-effectiveness, resources utilisation, and reduction on time or hospital admissions. Timeliness will be evaluated using data on waiting times, appointment availability, and speed to follow-up care. Patient-centredness, may involve both quantitative and qualitative assessment, depending on how studies report patient satisfaction; this could include satisfaction scores, survey, interviews. Safety will be analysed using quantitative data, including reported errors, adverse events, and other safety-related outcomes. Finally, for equity domain we will mainly rely on quantitative data on access disparities among different sociodemographic groups by age, ethnicity, or geographic location.
**19.** It is mentioned that the heterogeneity of outcome measures makes a meta-analysis unlikely. It would be helpful to explain what will be done to address this heterogeneity in the narrative analysis.	**19.** We recognise the potential for heterogeneity across studies. To address this, as clarified in the data extraction forms, we will categorise and synthesise the outcomes within each NAM domains, discussing variations in study populations, interventions, type or model of consultation (Video or telephone), which will allow us to provide a coherent narrative analysis.
**20.** As mentioned before a potential limitation is the lack of definition of the	**20.** We have provided the relevant details regarding this in our response to point **18** above.

NAM domains; the included studies may lack consistent definitions and measures, leading to possible challenges in comparing results so it would be helpful to explain what will be done to address this.	
**21.** Please provide a description of any subgroup analyses intended for the study. Subgroups may include analysis by year, age group of the patients, tyme from diagnosis, health status, pandemic time-lapse, use of artificial intelligence, new technologies, etc.	**21.** We have provided a description of intended subgroup analysis; "Subgroup analysis will be conducted based on factors such as different modalities of virtual consultations, country, settings, patient age group".
**22.** Justify the absence of the GRADE framework.	**22.** Due to the anticipated diversity of study designs and outcomes included in the review, we have decided not to include GRADE framework, instead we will focus on narrative synthesis and domain-specific analysis to evaluate the quality of evidence across the studies. As noted in the GRADE Handbook, the framework can be difficult to apply consistently across studies that differ significantly in design or methodology: https://training.cochrane.org/resource/grade-handbook
**23.** It would be beneficial to clarify how patient partners will be selected and involved in interpreting the results.	**23.** Thank you for your suggestion. We will liaise with the Patient and Public Involvement and Engagement (PPIE) theme at the Northwest London Applied Research Collaboration (NIHR) to ensure the selection of patient partners. We have added this clarification as a PPIE subheading in the manuscript as following:<br>"We will collaborate with the Patient and Public Involvement and Engagement (PPIE) theme at the Northwest London Applied Research Collaboration (NIHR) to ensure diverse representation in the review process. Selected patient partners will be actively involved in interpreting the results and provide insight into the findings."
**24.** There is contradiction in the concepts of synchronous and asynchronous times in Table 2 and in the intervention section, handle the explanation of this correctly.	**24.** Thank you for bringing our attention to this. We have corrected and clarified these in table 2 and intervention section.
**Regarding the funding**<br>**1.** Page 21 support, sources: It was stated that no financial support was received. However, in page 11 lines 20-22 it is mentioned that the work is supported by the NIHR.	**1.** Thank you for your observation. We have corrected the manuscript to reflect the funding support received from NIHR.
**2.** Page 11 lines 28-32 should go to the "declaration of interests" subtitle.	**2.** We moved lines 28-31 on page 11 to the "Declaration of Interests" section as suggested.
**Regarding the discussion:**<br><br>**1.** The discussion section helps us to contrasts the results of different studies with the results of the present one, although authors cite on the Introduction studies that helps us understand the magnitude of the issue, it would be helpful to also include similar studies that have assessed the quality of care of	**1.** Thank you for your suggestion. We have expand the discussion to include similar studies that have assessed the quality of care using NAM's domains, the edited version of the manuscript now reads as follows: "The use of NAM domains, a well-defined framework, provides a structured approach to evaluation. The application of this framework in other systematic reviews (32-34), where efficiency was measured by reduced appointment durations, effectiveness by improvement

consultations using the NAM ́s quality domains to understand how are they measured.	in HbA1c levels, and patient-centres through satisfaction surveys, demonstrates its robust utility in assessing the impact of digital interventions on care quality such as virtual consultations."
**2.** You mention the use of automatation tools as an strenght of the systematic review, but in the methods there is no description of which and how automatation tools will be utilized. Please provide a detailed description of this. Extra	**2.** After careful consideration we decided not to use the automated tool and use Covidence. We have updated the manuscript accordingly: "Title and abstract screening will be conducted using Covidence software The screening will be conducted in duplicate, with two reviewers independently assessing each study. For data extraction, one reviewer will extract the data, and the second reviewer will independently review the extracted data for accuracy and completeness. Any discrepancy will be resolved through discussion or by involving a third reviewer if necessary."
**3.** Please check for grammatical and typographical errors.	**3.** Thank you for pointing this out. We will carefully review the manuscript for grammatical and typographical errors.

VERSION 2 - AUTHOR RESPONSE

Reviewer comments	Response
*Please note the EMBASE search appears to be missing from supplementary I and Web of Science appears to be included twice. Please include this and/or amend the supplementary file.	Thank you for bringing this to our attention. We have updated Supplementary File I to include the EMBASE search strategy. The duplicate entry of Web of Science has been also corrected. The supplementary file now reflects all databases and search terms used.
*Please complete a thorough proofread of the text and correct any spelling and grammar errors that you identify, for example, "The included studies will required to have the traditional care of the same consultation delivered in-person."	We have conducted a proofreading of the manuscript to correct grammatical and typographical errors. All identified errors have been corrected now.
*We note the search will be conducted up to December 2023. Please ensure the search will be up to date (i.e., 2024). Please also update your PROSPERO record to indicate any new updated search limits.	We have changed our search timeline to include studies up to date. This change has been reflected throughout the Abstract and Methods sections. Our PROSPERO registration has also been updated to reflect the search limits.
*Please note that the Abstract section states the search will be conducted up until December 2023, yet the Methods section states that "studies published between January 2010 and up to date will be included". Please clarify the search dates and time limits and ensure these are consistent.	Thank you for highlighting this inconsistency. This has been consistently updated in both the Abstract and Methods to be from 2010 to 2014.
*Please amend the following statement, at present it is somewhat unclear: "If no response is received, the study will be included, but the	The review will follow PRISMA protocol, therefore, the missing data will be evaluated to determine whether it significantly affects the outcomes or conclusions. Based on this

missing data will be noted in the discussion and limitation.”	assessment, a decision will be made on whether to include or exclude the study. The updated manuscript now is read as:<br>“If no response is received, we will evaluate the potential impact of the missing data on. Studies with critical missing data that could significantly affect the analysis may be excluded. If a study is included despite incomplete data, the missing data will be noted in the limitation, and the risk of bias will be assessed accordingly. “
*Please include the planned start and end dates for the study in the methods section.	The planned start and end dates for the study have been added to the Methods section. The updated manuscript is now reads as follow:<br>“The review is planned to start on 31st of January 2024 and expected to be completed by 31st March 2025.”
*Please note the reviewer’s comment below has not been included in the revised manuscript:<br><br>The authors seemed not to specify report characteristics like language and publication status to be used as criteria for eligibility for the review.<br><br>We have now explicitly stated the language and publication status criteria in the manuscript to clarify the review process, The edited version of the manuscript now reads as follows:<br>“Inclusion criteria were not restricted by language. Non-English studies will be translated by professional services if selected for inclusion. Both peer-reviewed and grey literature will be included. Grey literature will be identified through Google Scholar and the criteria for inclusion will be consistent with the overall study selection criteria.”	We apologise for missing this. We have now stated the language publication status criteria in the Study selection criteria of the Methods. The edited version of the manuscript now reads as follows:<br>“Inclusion criteria were not restricted by language. Non-English studies will be translated by professional services if selected for inclusion.”
*Please note the reviewer’s comment below has not been included in the revised manuscript:<br><br>Since it seems to include all geographic locations and country income levels, it should also specify whether abstracts and articles not in English will be included (they should be, unless a very strong justification could be made).	We have clarified in the manuscript that we will include all languages, the edited version of the manuscript now reads as follows:<br>“Inclusion criteria are not restricted by language. Studies published in languages other than English will be included in the review”.

Thank you for your comment. We confirm that our review will include abstracts and articles from all geographic locations and country income levels, regardless of the language of publication. A clarification of this was added in the selection criteria as following: "Inclusion criteria are not restricted by language. Studies published in languages other than English will be included in the review"	
*Please clarify the reviewer's comment below in the manuscript and clearly describe how you will search for grey literature. Please also include any relevant search terms in supplementary file I.<br><br>Please describe where and how would you search for grey literature and what will be the criteria to include.<br><br>We have expanded our description of how and where grey literature will be searched, and criteria for inclusion.	We have expanded the Methods section to detail our grey literature search strategy. The edited version of the manuscript now reads as follows: "Grey literature will be identified through Google Scholar and the criteria for inclusion will be consistent with the overall study selection criteria."
*Please clarify the reviewer's comment below in further detail and justify this time limit in the manuscript:<br>In the search strategy, is there any reason to just use the last 10 years? Please justify it a little. We suggest using the time frame for the search change to 2013 up the date the search strategy is conducted.<br><br>Thank you for the suggestion. We decided to retain the search period from January 2010 and December 2023. The rational for this period is that the adoption of digital health technologies begun to accelerate around 2010, and the timeframe will include both pre- and post-pandemic studies, which will help to understand how virtual consultations have develop, we have provided this justification in the search strategy section of the protocol.	We have now updated the manuscript to include this justification and added the references, the revised version of the manuscript now reads as follows: "The rational for choosing the period from 2010 to 2023 is that the adoption of digital health technologies begun to accelerate around 2010, this period marks the beginning of widespread integration of electronic health records (EHR), telemedicine, and other digital innovations in healthcare systems(33, 34). This timeframe will include both pre- and post-pandemic studies, which will help to understand how virtual consultations have develop."
*Please address the reviewer's comment below in the manuscript:<br>It is unclear whether the six domains of the National Academy of Medicine (NAM) will be evaluated and retrieved as quantitative or qualitative data. I couldn't find a specific method for this on the Agency for HealthCare	Table 1 Mapping the impact of remote consultations for T2D patients based on the NAM* six domains analytical framework of quality of care, has been updated to present the variables more precisely. Examples have also been added to clarify the quantitative and qualitative measures.

Research and Quality page. This is particularly important because page nine mentions, 'The impact of each element within the domains will be statistically analyzed to demonstrate whether a negative, positive, or no impact is indicated.' Additionally, Table 1 indicates that domains like 'effective' and 'equitable' will be evaluated using quantitative measures. It is essential to more precisely define the domains, as Table 1 contains several instances of phrases like 'such as.' Remember, your analysis plan is crucial, so presenting your variables could also be helpful, although this will be discussed later on.<br><br>Thanks for bringing up this matter. The evaluation of the NAM domains will involve both quantitative and qualitative data. Domains such as effectiveness will be assessed using quantitative measures, including clinical outcomes (e.g., HbA1c levels, blood pressure, complications rate). Efficiency will also be assessed through quantitative metrics, such as cost-effectiveness, resources utilisation, and reduction on time or hospital admissions. Timeliness will be evaluated using data on waiting times, appointment availability, and speed to follow-up care. Patient-centredness, may involve both quantitative and qualitative assessment, depending on how studies report patient satisfaction; this could include satisfaction scores, survey, interviews. Safety will be analysed using quantitative data, including reported errors, adverse events, and other safety-related outcomes. Finally, for equity domain we will mainly rely on quantitative data on access disparities among different sociodemographic groups by age, ethnicity, or geographic location.	
*Please address the reviewer's comment below in the manuscript:<br>It is mentioned that the heterogeneity of outcome measures makes a meta-analysis unlikely. It would be helpful to explain what will be done to address this heterogeneity in the narrative analysis.<br><br>We recognise the potential for heterogeneity across studies. To address this, as clarified in the data extraction forms, we will categorise	The approaches to address the heterogeneity of outcome measures have been clarified in the Synthesis Strategy section of the Methods. We have updated this section to clarify the approaches for handling heterogeneity as applicable. The revised manuscript now reads as follows:<br>"To address the variability in study populations, interventions, and consultations modalities, results will be mapped across each of the NAM domains, with the key findings presented in a

and synthesise the outcomes within each NAM domains, discussing variations in study populations, interventions, type or model of consultation (Video or telephone), which will allow us to provide a coherent narrative analysis.	table for each domain. Descriptive statistics (means, medians, percentages) will be used to summarise quantitative data where appropriate, while subgroup analysis will be conducted based on consultation modalities (e.g., video vs. telephone), country, setting, and patient age, if the data allows. When applicable, inferential statistics will be used to compare outcomes between virtual and in-person consultations. The potential for long-term integration of virtual consultations will also be assessed if reported by the included studies.”
